# Ethyl (*Z*)-2-cyano-3-(9-ethyl-9*H*-carbazol-3-yl)prop-2-enoate

**DOI:** 10.1107/S1600536809015505

**Published:** 2009-04-30

**Authors:** Abdullah Mohamed Asiri, Mehmet Akkurt, Salman A. Khan, Islam Ullah Khan, Muhammad N. Arshad

**Affiliations:** a Chemistry Department, Faculty of Science, King Abdul-Aziz University, PO Box 80203, Jeddah 21589, Saudi Arabia; b Department of Physics, Faculty of Arts and Sciences, Erciyes University, 38039 Kayseri, Turkey; c Department of Chemistry, Government College University, Lahore, Pakistan

## Abstract

In the title compound, C_20_H_18_N_2_O_2_, weak inter­molecular C—H⋯O and C—H⋯N inter­actions generate a chain that runs parallel to the *b* axis and incorporates *C*(7) and *R*_2_^2^(15) graph-set motifs. The supra­molecular aggregation is completed by the presence of weak C—H⋯π inter­actions.

## Related literature

For background to the applications of carbazole derivatives, see: Park *et al.* (1998 ▶); Kimoto *et al.* (2004 ▶). For reference structural data, see: Allen *et al.* (1987 ▶).
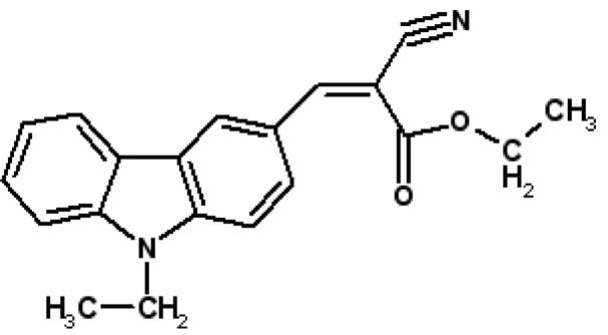


## Experimental

### 

#### Crystal data

C_20_H_18_N_2_O_2_
*M* *_r_* = 318.36
Monoclinic, 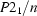

*a* = 10.8030 (7) Å
*b* = 13.4443 (10) Å
*c* = 11.6160 (7) Å
β = 93.387 (5)°
*V* = 1684.15 (19) Å^3^
*Z* = 4
Mo *K*α radiation
μ = 0.08 mm^−1^
*T* = 296 K
0.35 × 0.10 × 0.05 mm

#### Data collection

Bruker SMART CCD diffractometer
Absorption correction: none
19261 measured reflections
4195 independent reflections
1121 reflections with *I* > 2σ(*I*)
*R*_int_ = 0.146

#### Refinement

*R*[*F* ^2^ > 2σ(*F* ^2^)] = 0.084
*wR*(*F* ^2^) = 0.228
*S* = 0.91
4195 reflections
221 parameters
H-atom parameters constrained
Δρ_max_ = 0.32 e Å^−3^
Δρ_min_ = −0.23 e Å^−3^

### 

Data collection: *SMART* (Bruker, 2003 ▶); cell refinement: *SAINT-Plus* (Bruker, 2003 ▶); data reduction: *SAINT-Plus*; program(s) used to solve structure: *SIR97* (Altomare *et al.*, 1999 ▶); program(s) used to refine structure: *SHELXL97* (Sheldrick, 2008 ▶); molecular graphics: *ORTEP-3* (Farrugia, 1997 ▶); software used to prepare material for publication: *WinGX* (Farrugia, 1999 ▶) and *PLATON* (Spek, 2009 ▶).

## Supplementary Material

Crystal structure: contains datablocks global, I. DOI: 10.1107/S1600536809015505/hb2957sup1.cif

Structure factors: contains datablocks I. DOI: 10.1107/S1600536809015505/hb2957Isup2.hkl

Additional supplementary materials:  crystallographic information; 3D view; checkCIF report

## Figures and Tables

**Table 1:** Hydrogen-bond geometry (Å, °)

*D*—H⋯*A*	*D*—H	H⋯*A*	*D*⋯*A*	*D*—H⋯*A*
C9—H9⋯O1^i^	0.93	2.58	3.255 (5)	130
C12—H12⋯N2^ii^	0.93	2.58	3.491 (6)	165
C17—H17*B*⋯*Cg*2^iii^	0.97	2.82	3.692 (5)	150

Symmetry codes: (i) 
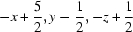
; (ii) 
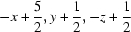
; (iii) 
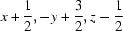
. *Cg*2 is the centroid of the C7–C12 ring.

**Fig. 1.**
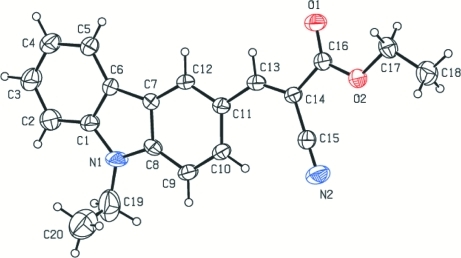
View of (I), showing 30% displacement ellipsoids for the non-hydrogen atoms.

**Fig. 2.**
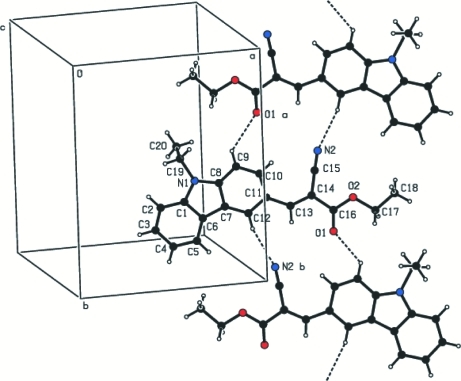
View of the unit cell of (I), viewed along the c axis, showing the network of hydrogen bonds.

## supplementary crystallographic information

### Comment

Nonlinear optical (NLO) andelectro-optic (EO) properties of organic dyes have been the hot subject nowadays because of the potential applications in optical switching, optical telecommunication devices, optical disks, new type of dye lasers (Kimoto *et al.*, 2004). Carbazole derivatives have important roles of optical material due to their special photorefractive, electrical, and chemical properties. Carbazoles are well known as a conjugated, good hole transporting, electron-donor, planar compound and easy to introduce solubilizing groups to rigid ring structure (Park *et al.*, 1998).

The molecular structure of title compound (I) is shown in Fig. 1. The bond lengths and angles in (I) display normal values (Allen *et al.*, 1987). The nine-membered ring N1/C1—C8 is essentially planar, with maximum deviations of 0.014 (5)Åfor C5 and -0.025 (4) Å for C7 from its mean plane, respectively. In the molecule of (I), the rest atoms of the molecule lie close to the nine-membered ring plane, with the maximum deviations of 1.110 (7), -0.467 (3) and -0.341 (5) Å, for C20, O1 and C18, respectively.

The crystal structure of (I) is stabilized by weak C—H···O and C—H···N interactions (Table 1, Fig. 2), that generates a chain which runs parallel to the *b*axis and has the graph-set motifs of *C*(7) and *R*_2_^2^(15). The supramolecular aggregation is completed by the presence of C—H···π interactions (Table 1).

### Experimental

Equivalent molar quantities of *N*-ethyl carbazol-9-carboxaldehyde (1.0 g, 4.48 mmol) and ethylcyanoacetate (0.51 g, 4.48 mmol) were dissolved in 25 ml e thanol then heated at reflux. Pipyridine (one drop) was added to the solution and reflux was continued for 6 h. The solution was cooled to room temperature and the solid products were filtered and washed with ethanol (25 ml). Recrystalization from ethanol gave yellow prisms of (I). Yield: (0.4 g, 29%); m.p. 385 K; IR (KBr) ν_max _cm^-1^. 3028 (C—H aromatic), 2978 (–C—H aliphatic), 2216 (CN), 1721(C=O), 1573 (C=C), 1225 (C—O), 1127 (C—N).). ^1^H NMR(CDCl_3_): δ 8.15 (H-1), 8.19 (H-2), 8.41(H-3), 7.45 (H-4), 7.33 (H-5), 7.54 (H-6), 7.45 (H-7), 8.74 (H-8), 4.39 (CH_3_—CH_2_—N), 1.41 (CH_3_—CH_2_—N), 4.39 (CH_3_—CH_2_—O), 1.41 (CH_3_—CH_2_—O).

### Refinement

All H atoms were positioned geometrically with C—H = 0.93–0.97 Å and refined as riding with *U*_iso_(H) = 1.2 or 1.5*U*_eq_(C).

### Crystal data

**Table:**

C_20_H_18_N_2_O_2_	*F*(000) = 672
*M**_r_* = 318.36	*D*_x_ = 1.256 Mg m^−^^3^
Monoclinic, *P*2_1_/*n*	Mo *K*α radiation, λ = 0.71073 Å
Hall symbol: -P 2yn	Cell parameters from 1000 reflections
*a* = 10.8030 (7) Å	θ = 2.3–18.8°
*b* = 13.4443 (10) Å	µ = 0.08 mm^−^^1^
*c* = 11.6160 (7) Å	*T* = 296 K
β = 93.387 (5)°	Prism, yellow
*V* = 1684.15 (19) Å^3^	0.35 × 0.10 × 0.05 mm
*Z* = 4	

### Data collection

**Table:**

Bruker SMART CCD diffractometer	1121 reflections with *I* > 2σ(*I*)
Radiation source: sealed tube	*R*_int_ = 0.146
graphite	θ_max_ = 28.4°, θ_min_ = 2.3°
ω scans	*h* = −14→14
19261 measured reflections	*k* = −17→17
4195 independent reflections	*l* = −15→15

### Refinement

**Table:**

Refinement on *F*^2^	Primary atom site location: structure-invariant direct methods
Least-squares matrix: full	Secondary atom site location: difference Fourier map
*R*[*F*^2^ > 2σ(*F*^2^)] = 0.084	Hydrogen site location: inferred from neighbouring sites
*wR*(*F*^2^) = 0.228	H-atom parameters constrained
*S* = 0.91	*w* = 1/[σ^2^(*F*_o_^2^) + (0.0844*P*)^2^] where *P* = (*F*_o_^2^ + 2*F*_c_^2^)/3
4195 reflections	(Δ/σ)_max_ = 0.001
221 parameters	Δρ_max_ = 0.32 e Å^−^^3^
0 restraints	Δρ_min_ = −0.23 e Å^−^^3^

### Special details

**Table:**

Geometry. Bond distances, angles *etc*. have been calculated using the rounded fractional coordinates. All su's are estimated from the variances of the (full) variance-covariance matrix. The cell e.s.d.'s are taken into account in the estimation of distances, angles and torsion angles
Refinement. Refinement on *F*^2^ for ALL reflections except those flagged by the user for potential systematic errors. Weighted *R*-factors *wR* and all goodnesses of fit *S* are based on *F*^2^, conventional *R*-factors *R* are based on *F*, with *F* set to zero for negative *F*^2^. The observed criterion of *F*^2^ > σ(*F*^2^) is used only for calculating -*R*-factor-obs *etc*. and is not relevant to the choice of reflections for refinement. *R*-factors based on *F*^2^ are statistically about twice as large as those based on *F*, and *R*-factors based on ALL data will be even larger.

### Fractional atomic coordinates and isotropic or equivalent isotropic displacement parameters (Å^2^)

**Table:**

	*x*	*y*	*z*	*U*_iso_*/*U*_eq_	
O1	1.4145 (3)	0.8481 (2)	0.1597 (3)	0.0835 (14)	
O2	1.5181 (3)	0.70597 (19)	0.1392 (2)	0.0621 (12)	
N1	0.7557 (3)	0.6264 (3)	0.3838 (4)	0.0812 (19)	
N2	1.3601 (4)	0.5077 (3)	0.2087 (4)	0.102 (2)	
C1	0.7049 (4)	0.7186 (3)	0.4082 (4)	0.0608 (19)	
C2	0.5892 (4)	0.7421 (4)	0.4444 (4)	0.085 (2)	
C3	0.5636 (4)	0.8400 (4)	0.4611 (4)	0.087 (3)	
C4	0.6497 (5)	0.9127 (4)	0.4426 (4)	0.079 (2)	
C5	0.7649 (4)	0.8899 (3)	0.4064 (4)	0.0641 (19)	
C6	0.7937 (4)	0.7906 (3)	0.3871 (3)	0.0502 (17)	
C7	0.9004 (4)	0.7407 (3)	0.3476 (3)	0.0483 (17)	
C8	0.8738 (4)	0.6380 (3)	0.3497 (4)	0.0571 (17)	
C9	0.9580 (4)	0.5671 (3)	0.3175 (4)	0.0713 (19)	
C10	1.0695 (4)	0.5990 (3)	0.2815 (3)	0.0613 (19)	
C11	1.0991 (4)	0.7019 (3)	0.2751 (3)	0.0520 (17)	
C12	1.0125 (4)	0.7700 (3)	0.3092 (3)	0.0520 (16)	
C13	1.2138 (4)	0.7387 (3)	0.2355 (3)	0.0544 (16)	
C14	1.3189 (4)	0.6959 (3)	0.2050 (3)	0.0526 (17)	
C15	1.3401 (4)	0.5905 (4)	0.2063 (4)	0.0690 (19)	
C16	1.4198 (4)	0.7584 (3)	0.1657 (4)	0.0579 (17)	
C17	1.6223 (4)	0.7618 (3)	0.1012 (4)	0.0669 (17)	
C18	1.7166 (4)	0.6885 (4)	0.0645 (4)	0.088 (2)	
C19	0.6800 (6)	0.5253 (5)	0.3793 (6)	0.131 (3)	
C20	0.6995 (6)	0.4966 (5)	0.4914 (6)	0.148 (4)	
H2	0.53090	0.69290	0.45680	0.1020*	
H3	0.48630	0.85800	0.48560	0.1040*	
H4	0.62930	0.97890	0.45490	0.0950*	
H5	0.82260	0.93980	0.39510	0.0770*	
H9	0.93920	0.49970	0.32020	0.0860*	
H10	1.12760	0.55220	0.26070	0.0740*	
H12	1.03080	0.83750	0.30600	0.0620*	
H13	1.21550	0.80770	0.22980	0.0650*	
H17A	1.65730	0.80300	0.16350	0.0800*	
H17B	1.59600	0.80470	0.03720	0.0800*	
H18A	1.74330	0.64750	0.12900	0.1320*	
H18B	1.78650	0.72350	0.03720	0.1320*	
H18C	1.68040	0.64740	0.00390	0.1320*	
H19A	0.59270	0.53590	0.35850	0.1570*	
H19B	0.71370	0.47770	0.32670	0.1570*	
H20A	0.78660	0.48700	0.50880	0.2220*	
H20B	0.65620	0.43550	0.50350	0.2220*	
H20C	0.66930	0.54720	0.54090	0.2220*	

### Atomic displacement parameters (Å^2^)

**Table:**

	*U*^11^	*U*^22^	*U*^33^	*U*^12^	*U*^13^	*U*^23^
O1	0.078 (2)	0.046 (2)	0.130 (3)	−0.0031 (17)	0.0351 (19)	0.0023 (19)
O2	0.051 (2)	0.056 (2)	0.081 (2)	−0.0006 (16)	0.0186 (16)	0.0005 (15)
N1	0.066 (3)	0.039 (3)	0.142 (4)	−0.016 (2)	0.036 (2)	0.001 (2)
N2	0.092 (3)	0.052 (3)	0.165 (5)	0.015 (2)	0.042 (3)	0.006 (3)
C1	0.057 (3)	0.043 (3)	0.084 (4)	−0.004 (2)	0.017 (3)	−0.003 (2)
C2	0.058 (3)	0.071 (4)	0.128 (5)	−0.001 (3)	0.029 (3)	0.000 (3)
C3	0.062 (4)	0.077 (4)	0.124 (5)	0.016 (3)	0.027 (3)	−0.005 (3)
C4	0.074 (4)	0.059 (3)	0.107 (4)	0.014 (3)	0.021 (3)	−0.002 (3)
C5	0.060 (3)	0.054 (3)	0.080 (4)	0.010 (2)	0.019 (3)	0.002 (2)
C6	0.050 (3)	0.045 (3)	0.057 (3)	0.005 (2)	0.015 (2)	0.000 (2)
C7	0.052 (3)	0.043 (3)	0.051 (3)	−0.003 (2)	0.012 (2)	0.000 (2)
C8	0.049 (3)	0.046 (3)	0.078 (3)	−0.015 (2)	0.019 (2)	0.001 (2)
C9	0.065 (3)	0.036 (3)	0.116 (4)	−0.001 (3)	0.032 (3)	−0.004 (2)
C10	0.060 (3)	0.039 (3)	0.087 (4)	0.002 (2)	0.022 (3)	−0.006 (2)
C11	0.051 (3)	0.041 (3)	0.065 (3)	−0.004 (2)	0.012 (2)	0.000 (2)
C12	0.059 (3)	0.034 (2)	0.064 (3)	−0.003 (2)	0.013 (2)	−0.001 (2)
C13	0.059 (3)	0.038 (2)	0.067 (3)	−0.003 (2)	0.010 (2)	0.002 (2)
C14	0.050 (3)	0.039 (3)	0.070 (3)	0.001 (2)	0.014 (2)	−0.001 (2)
C15	0.059 (3)	0.056 (3)	0.095 (4)	0.004 (3)	0.031 (3)	0.001 (3)
C16	0.052 (3)	0.050 (3)	0.073 (3)	0.000 (3)	0.016 (2)	0.000 (2)
C17	0.050 (3)	0.074 (3)	0.078 (3)	−0.009 (3)	0.014 (3)	0.003 (3)
C18	0.068 (3)	0.094 (4)	0.103 (4)	0.012 (3)	0.020 (3)	0.009 (3)
C19	0.123 (5)	0.164 (7)	0.110 (6)	0.062 (5)	0.041 (5)	0.026 (5)
C20	0.156 (7)	0.134 (6)	0.156 (8)	0.028 (5)	0.023 (6)	−0.008 (5)

### Geometric parameters (Å, °)

**Table:**

O1—C16	1.209 (5)	C14—C15	1.435 (7)
O2—C16	1.326 (5)	C14—C16	1.470 (6)
O2—C17	1.444 (5)	C17—C18	1.497 (6)
N1—C1	1.392 (6)	C19—C20	1.363 (10)
N1—C8	1.367 (6)	C2—H2	0.9300
N1—C19	1.586 (8)	C3—H3	0.9300
N2—C15	1.134 (7)	C4—H4	0.9300
C1—C2	1.379 (6)	C5—H5	0.9300
C1—C6	1.395 (6)	C9—H9	0.9300
C2—C3	1.361 (8)	C10—H10	0.9300
C3—C4	1.375 (7)	C12—H12	0.9300
C4—C5	1.372 (7)	C13—H13	0.9300
C5—C6	1.392 (6)	C17—H17A	0.9700
C6—C7	1.432 (6)	C17—H17B	0.9700
C7—C8	1.411 (6)	C18—H18A	0.9600
C7—C12	1.373 (6)	C18—H18B	0.9600
C8—C9	1.384 (6)	C18—H18C	0.9600
C9—C10	1.367 (6)	C19—H19A	0.9700
C10—C11	1.423 (6)	C19—H19B	0.9700
C11—C12	1.384 (6)	C20—H20A	0.9600
C11—C13	1.435 (6)	C20—H20B	0.9600
C13—C14	1.339 (6)	C20—H20C	0.9600
			
C16—O2—C17	116.4 (3)	C3—C2—H2	121.00
C1—N1—C8	110.1 (4)	C2—C3—H3	119.00
C1—N1—C19	124.2 (4)	C4—C3—H3	119.00
C8—N1—C19	125.2 (4)	C3—C4—H4	119.00
N1—C1—C2	129.9 (4)	C5—C4—H4	119.00
N1—C1—C6	107.4 (4)	C4—C5—H5	121.00
C2—C1—C6	122.7 (4)	C6—C5—H5	121.00
C1—C2—C3	117.4 (4)	C8—C9—H9	121.00
C2—C3—C4	121.3 (4)	C10—C9—H9	121.00
C3—C4—C5	121.6 (5)	C9—C10—H10	119.00
C4—C5—C6	118.6 (4)	C11—C10—H10	119.00
C1—C6—C5	118.3 (4)	C7—C12—H12	119.00
C1—C6—C7	107.8 (4)	C11—C12—H12	119.00
C5—C6—C7	133.8 (4)	C11—C13—H13	113.00
C6—C7—C8	106.5 (4)	C14—C13—H13	113.00
C6—C7—C12	135.4 (4)	O2—C17—H17A	110.00
C8—C7—C12	118.1 (4)	O2—C17—H17B	110.00
N1—C8—C7	108.1 (4)	C18—C17—H17A	110.00
N1—C8—C9	129.7 (4)	C18—C17—H17B	110.00
C7—C8—C9	122.1 (4)	H17A—C17—H17B	108.00
C8—C9—C10	118.1 (4)	C17—C18—H18A	109.00
C9—C10—C11	121.7 (4)	C17—C18—H18B	109.00
C10—C11—C12	118.1 (4)	C17—C18—H18C	109.00
C10—C11—C13	123.6 (4)	H18A—C18—H18B	109.00
C12—C11—C13	118.4 (4)	H18A—C18—H18C	109.00
C7—C12—C11	121.8 (4)	H18B—C18—H18C	110.00
C11—C13—C14	134.3 (4)	N1—C19—H19A	112.00
C13—C14—C15	124.0 (4)	N1—C19—H19B	112.00
C13—C14—C16	119.5 (4)	C20—C19—H19A	112.00
C15—C14—C16	116.5 (4)	C20—C19—H19B	112.00
N2—C15—C14	178.1 (5)	H19A—C19—H19B	110.00
O1—C16—O2	123.6 (4)	C19—C20—H20A	109.00
O1—C16—C14	123.7 (4)	C19—C20—H20B	109.00
O2—C16—C14	112.8 (3)	C19—C20—H20C	109.00
O2—C17—C18	107.5 (3)	H20A—C20—H20B	110.00
N1—C19—C20	99.3 (5)	H20A—C20—H20C	109.00
C1—C2—H2	121.00	H20B—C20—H20C	109.00
			
C17—O2—C16—O1	−0.2 (6)	C1—C6—C7—C12	176.6 (4)
C17—O2—C16—C14	−179.2 (3)	C5—C6—C7—C8	178.3 (4)
C16—O2—C17—C18	−174.2 (4)	C5—C6—C7—C12	−3.3 (8)
C8—N1—C1—C2	179.6 (5)	C6—C7—C8—N1	2.4 (5)
C8—N1—C1—C6	0.8 (5)	C6—C7—C8—C9	−179.4 (4)
C19—N1—C1—C2	7.6 (8)	C12—C7—C8—N1	−176.4 (4)
C19—N1—C1—C6	−171.1 (5)	C12—C7—C8—C9	1.8 (6)
C1—N1—C8—C7	−2.1 (5)	C6—C7—C12—C11	−179.5 (4)
C1—N1—C8—C9	180.0 (5)	C8—C7—C12—C11	−1.1 (6)
C19—N1—C8—C7	169.8 (5)	N1—C8—C9—C10	177.0 (5)
C19—N1—C8—C9	−8.2 (8)	C7—C8—C9—C10	−0.8 (7)
C1—N1—C19—C20	−90.6 (6)	C8—C9—C10—C11	−0.9 (6)
C8—N1—C19—C20	98.7 (6)	C9—C10—C11—C12	1.5 (6)
N1—C1—C2—C3	−179.5 (5)	C9—C10—C11—C13	−178.6 (4)
C6—C1—C2—C3	−0.9 (7)	C10—C11—C12—C7	−0.4 (5)
N1—C1—C6—C5	−179.4 (4)	C13—C11—C12—C7	179.7 (3)
N1—C1—C6—C7	0.7 (5)	C10—C11—C13—C14	−5.7 (7)
C2—C1—C6—C5	1.7 (6)	C12—C11—C13—C14	174.2 (4)
C2—C1—C6—C7	−178.2 (4)	C11—C13—C14—C15	−0.1 (7)
C1—C2—C3—C4	0.0 (7)	C11—C13—C14—C16	179.0 (4)
C2—C3—C4—C5	0.1 (7)	C13—C14—C16—O1	0.6 (7)
C3—C4—C5—C6	0.7 (7)	C13—C14—C16—O2	179.6 (3)
C4—C5—C6—C1	−1.5 (6)	C15—C14—C16—O1	179.7 (4)
C4—C5—C6—C7	178.3 (4)	C15—C14—C16—O2	−1.3 (5)
C1—C6—C7—C8	−1.9 (4)		

### Hydrogen-bond geometry (Å, °)

**Table:**

*D*—H···*A*	*D*—H	H···*A*	*D*···*A*	*D*—H···*A*
C9—H9···O1^i^	0.93	2.58	3.255 (5)	130
C12—H12···N2^ii^	0.93	2.58	3.491 (6)	165
C17—H17B···Cg2^iii^	0.97	2.82	3.692 (5)	150

Symmetry codes: (i) −*x*+5/2, *y*−1/2, −*z*+1/2; (ii) −*x*+5/2, *y*+1/2, −*z*+1/2; (iii) *x*+1/2, −*y*+3/2, *z*−1/2.
